# Experimental crescentic glomerulonephritis: a new bicongenic rat model

**DOI:** 10.1242/dmm.012328

**Published:** 2013-08-15

**Authors:** Zelpha D’Souza, Stephen P. McAdoo, Jennifer Smith, Charles D. Pusey, H. Terence Cook, Jacques Behmoaras, Timothy J. Aitman

**Affiliations:** 1 MRC Clinical Sciences Centre, Imperial College London, Hammersmith Hospital, Du Cane Road, London, W12 0NN, UK; 2 Renal and Vascular Inflammation Section, Imperial College London, Hammersmith Hospital, Du Cane Road, London, W12 0NN, UK; 3 Centre for Complement and Inflammation Research, Imperial College London, Hammersmith Hospital, Du Cane Road, London, W12 0NN, UK

## Abstract

Crescentic glomerulonephritis (CRGN) is a major cause of human kidney failure, but the underlying mechanisms are not fully understood. Wistar Kyoto (WKY) rats are uniquely susceptible to CRGN following injection of nephrotoxic serum, whereas Lewis (LEW) rats are resistant. Our previous genetic studies of nephrotoxic nephritis (NTN), a form of CRGN induced by nephrotoxic serum, identified *Fcgr3* and *Jund* as WKY genes underlying the two strongest quantitative trait loci for NTN phenotypes: *Crgn1* and *Crgn2*, respectively. We also showed that introgression of WKY *Crgn1* or *Crgn2* individually into a LEW background did not lead to the formation of glomerular crescents. We have now generated a bicongenic strain, LEW.W*Crgn1,2*, in which WKY *Crgn1* and C*rgn2* are both introgressed into the LEW genetic background. These rats show development of NTN phenotypes, including glomerular crescents. Furthermore, we characterised macrophage function and glomerular cytokine profiles in this new strain. Additionally, we show that LEW.W*Crgn1,2* rats are resistant to the development of glomerular crescents that is usually induced following immunisation with recombinant rat α3(IV)NC1, the specific Goodpasture autoantigen located in the glomerular basement membrane against which the immune response is directed in experimental autoimmune glomerulonephritis. Our results show that the new bicongenic strain responds differently to two distinct experimental triggers of CRGN. This is the first time that CRGN has been induced on a normally resistant rat genetic background and identifies the LEW.W*Crgn1,2* strain as a new, potentially valuable model of macrophage-dependent glomerulonephritis.

## INTRODUCTION

Glomerulonephritis (GN) is a major cause of human kidney failure, with the formation of glomerular epithelial crescents being a common feature in its most severe forms. Crescentic glomerulonephritis (CRGN) is characterised by the appearance of glomerular crescents formed by the accumulation of inflammatory cells and proliferating epithelial cells in Bowman’s space. Untreated CRGN most often leads to rapidly progressive glomerulonephritis followed by end-stage renal disease (Feehally et al., 2005).

The Wistar Kyoto (WKY) rat strain is highly susceptible to experimental models of CRGN, including nephrotoxic nephritis (NTN) and experimental autoimmune glomerulonephritis (EAG) (Reynolds et al., 2003; Tam et al., 1999). The WKY models of NTN and EAG closely resemble human CRGN histologically (Reynolds et al., 2003; Tam et al., 1999; Tarzi et al., 2011). NTN has been used widely as a model for studying mechanisms of crescent formation and factors leading to glomerulosclerosis and renal failure in CRGN (Aitman et al., 2006; Behmoaras et al., 2008; Behmoaras et al., 2010; Cook et al., 1999; Smith et al., 2007; Tam et al., 1999), whereas EAG has been used as a model of autoantibody production and autoimmune glomerular injury (Reynolds et al., 2012; Reynolds et al., 2002; Reynolds et al., 2003; Ryan et al., 2001). In the WKY rat, a single injection of nephrotoxic serum (NTS) leads to proteinuria, glomerular macrophage infiltration and glomerular crescent formation in 90% of glomeruli with progression to severe scarring with renal failure by week 6 (Behmoaras et al., 2008; Cook et al., 1999; Tam et al., 1999), whereas rat strains such as Lewis (LEW) and Brown Norway are resistant. The LEW strain shares the same MHC haplotype (RT1^l^) but shows resistance to CRGN following NTS and has therefore been used as a negative control in CRGN (Aitman et al., 2006; Behmoaras et al., 2008; Behmoaras et al., 2010; Maratou et al., 2011; Page et al., 2012; Smith et al., 2007).

Previously, we studied the genetic susceptibility to NTN in the WKY rat by using segregating populations derived from WKY and LEW rats. Genome-wide linkage analysis carried out on (WKY × LEW) F_2_ offspring detected seven significant quantitative trait loci (QTLs) for CRGN susceptibility (Aitman et al., 2006). Of these, two major QTLs, *Crgn1* and *Crgn2* (logarithm of odds >8), were mapped to chromosome 13 and chromosome 16, respectively.

EAG is a distinct model of crescentic nephritis that, rather than relying on passive transfer of heterologous nephrotoxic antibodies raised in another species, requires induction of autoimmunity to the glomerular basement membrane (GBM). In our laboratory, this is achieved by immunising rats with recombinant non-collagenous domain of the alpha 3 chain of type IV collagen [α3(IV)NC1], the Goodpasture autoantigen, which results in the development of circulating and deposited autoantibodies to this component of the GBM, and consequently CRGN (Ryan et al., 2001). As observed in NTN, the WKY rat strain is exquisitely susceptible to the induction of EAG, and the LEW strain is resistant (Reynolds et al., 2003). In contrast to NTN, however, the first generation (F_1_; WKY × LEW) remain resistant to the development of EAG, whereas F_1_ animals backcrossed to the parental WKY strain (BC_1_; WKY × F_1_) show a range of disease responses (Reynolds et al., 2002). These observations suggest that susceptibility to EAG is inherited as a complex trait distinct from susceptibility to NTN, although a recent genome-wide linkage analysis of this BC_1_ population revealed a QTL on chromosome 13, again corresponding to *Crgn1*, linked to the percentage of glomerular crescents in EAG, suggesting at least some shared genetic predisposition (Reynolds et al., 2012).

**RESOURCE IMPACT**

**Background**

Crescentic glomerulonephritis (CRGN) is a major cause of loss of kidney function, which, if left untreated, can lead to fatal renal failure. The histological features of CRGN, notably the presence of crescent-shaped scars in the glomeruli, are widely recognised; however, the molecular mechanisms underlying the disease are not completely understood. The rat has proven to be an ideal model for studies of different forms of CRGN, because the resulting histology closely resembles that of human CRGN and the molecular phenotypes are largely reproducible. In contrast, mouse models of CRGN suffer drawbacks, namely that the experiments are difficult to standardise and preimmunisation of mice is required. Among the commonly used experimental rat strains, the Wistar Kyoto (WKY) rat strain is susceptible to induction of CRGN, whereas the Lewis (LEW) rat strain is resistant. Two genetic loci (*Crgn1* and *Crgn2*) that are linked to CRGN susceptibility have been identified and congenic strains (strains that carry a specific genomic region from another strain; the remainder of their genome is their own) carrying *Crgn1* and/or *Crgn2* in either the WKY or LEW genetic background have been generated. These reciprocal congenic strains are valuable resources for studying the effect of these loci on susceptibility to CRGN. Introgressing LEW *Crgn1* and *Crgn2* loci into the WKY (CRGN-susceptible) genome has been shown to reduce the incidence of disease, but the effects exerted by both WKY *Crgn1* and *Crgn2* in a LEW (CRGN-resistant) genome have not been previously investigated.

**Results**

The authors generated a new bicongenic rat strain (LEW.W*Crgn1,2*) by introgressing CRGN-susceptible WKY *Crgn1* and *Crgn2* into the genetic background of the CRGN-resistant LEW strain. Two distinct forms of CRGN, nephrotoxic nephritis (NTN) and experimental autoimmune glomerulonephritis (EAG), were induced in LEW.W*Crgn1,2* rats via passive transfer of heterologous nephrotoxic antibodies raised in another species or induction of autoimmunity to the glomerular basement membrane, respectively. In the NTN model, the introgression of WKY *Crgn1* and *Crgn2* resulted in disease onset and increased macrophage activity in the normally CRGN-resistant LEW strain. In addition, the gene expression profiles for glomerular cytokines were significantly different in the bicongenic rat strain affected by NTN compared with the parental LEW strain. Interestingly, the new bicongenic rat strain did not show signs of disease in the EAG model, indicating that resistance to this form of CRGN is retained.

**Implications and future directions**

These results indicate differential responses of the new bicongenic rat strain to the induction of NTN and EAG, suggesting that different mechanisms drive these distinct forms of CRGN. The study substantiates previous evidence that macrophage activation is an important aspect of disease development, and provides new insights into the genes and pathways involved in macrophage-dependent CRGN. This is the first time that CRGN has been successfully induced in the normally resistant LEW strain; the resulting experimental model could be useful for the identification of additional CRGN-associated loci. Furthermore, the model could enable dissection of the mechanisms underlying progression from serological autoimmunity to glomerular damage and EAG, to which the strain is resistant.

Here, we show for the first time that the CRGN-resistant LEW rat strain develops glomerulonephritis, after a single dose of NTS, when both *Crgn1* and *Crgn2* from the CRGN-susceptible WKY are introgressed into its genetic background. We show that the presence of both loci is necessary to promote glomerular crescent formation in this bicongenic strain (LEW.W*Crgn1,2*). We also show that *Crgn1* and *Crgn2* alter the expression of key inflammatory cytokines in nephritic glomeruli following NTN induction. In LEW.W*Crgn1,2* bone-marrow-derived macrophages (BMDMs), Fc-receptor-mediated phagocytosis and gene expression differ significantly from the LEW background. Despite showing susceptibility to NTN, the new LEW.*WCrgn1,2* strain is resistant to glomerular autoantibody deposition and to EAG. These results demonstrate the importance of macrophage function associated with CRGN and identify the LEW.W*Crgn1,2* as a new model of macrophage-dependent CRGN.

## RESULTS

### LEW.W*Crgn1,2* rats show susceptibility to NTN

**Fig. 1.**
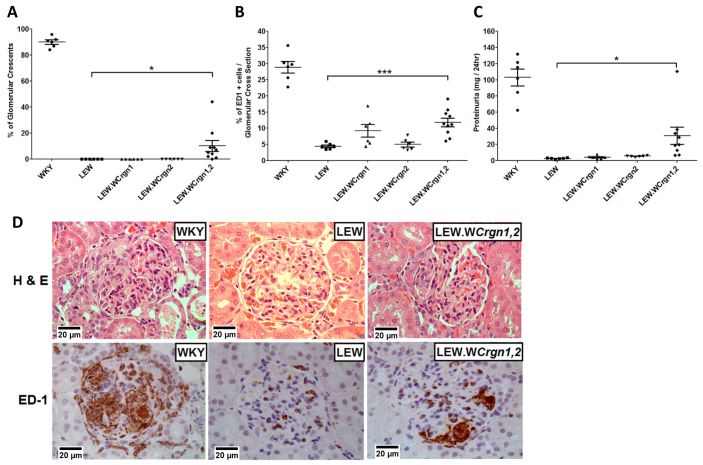
**NTN phenotypes in parental and congenic rat strains.** (A) Percentage of glomeruli with crescent formation. (B) Macrophage infiltration quantified by the percentage of ED1+ cells per glomerular cross-section. (C) Measurement of proteinuria. ***P<0.001; *P<0.05 between LEW.W*Crgn1,2* and LEW rats using oneway ANOVA with Newman-Keuls comparison test. WKY, LEW, LEW.W*Crgn1*, LEW.W*Crgn2* rats, *n*=6; LEW.W*Crgn1,2* rats, *n*=10, except for proteinuria, where *n*=9. (D) H&E staining (upper panels) and ED-1 (CD68) immunohistochemistry (lower panels) of glomeruli 10 days after injection with NTS. The H&E stain showed crescentic glomeruli in WKY rats and, for the first time, formation of glomerular crescents in a LEW-derived strain (LEW.*WCrgn1,2*), whereas LEW rats were resistant to crescent formation. ED-1 immunohistochemistry also showed a significant influx of glomerular macrophages in the LEW.W*Crgn1,2* strain.

NTN was induced in the WKY, LEW, single congenic (LEW.W*Crgn1* and LEW.W*Crgn2*) (*n*=6/group) and bicongenic (LEW.W*Crgn1,2; n*=10) rat strains. After 10 days, glomerular crescents were observed in 90±1.6% of glomeruli in WKY rats, 0% of glomeruli in LEW, LEW.W*Crgn1* and LEW.W*Crgn2* rats, and, for the first time on a normally NTN-resistant genetic background, introgression of WKY *Crgn1* and *Crgn2* in LEW rats resulted in 10±4.1% of glomerular crescent formation (Fig. 1A). In conjunction with glomerular crescent formation, prominent infiltration of macrophages into the glomeruli was observed in WKY rats [29±1.7% per glomerular cross section (gcs)], whereas this was minimal in LEW glomeruli (4±0.4% per gcs), LEW.W*Crgn1* glomeruli (9±1.9% per gcs) and LEW.W*Crgn2* glomeruli (5±0.7% per gcs). There was, however, a statistically significant increase in macrophage infiltration into the glomeruli of the LEW.W*Crgn1,2* strain (12±1.2% per gcs; Fig. 1B). In addition, proteinuria levels were measured in the parental WKY and LEW strains as well as in the LEW.W*Crgn1,2* strain and single congenic strains. Although differences in proteinuria were significant between the parental LEW and WKY strains, as previously shown (Aitman et al., 2006; Behmoaras et al., 2008), there was no significant difference in proteinuria levels between the LEW and the single congenic strains. There was, however, a highly significant increase in proteinuria in the LEW.W*Crgn1,2* rats compared with LEW rats (Fig. 1C). Glomerular crescent formation and macrophage infiltration are illustrated with haematoxylin and eosin (H&E) and immunohistochemical staining for ED-1-positive cells per gcs, respectively (Fig. 1D).

### Expression of *Tnfα*, *Nos2* and *Mmp12* in glomeruli from LEW.W*Crgn1,2* rats following NTN

**Fig. 2.**
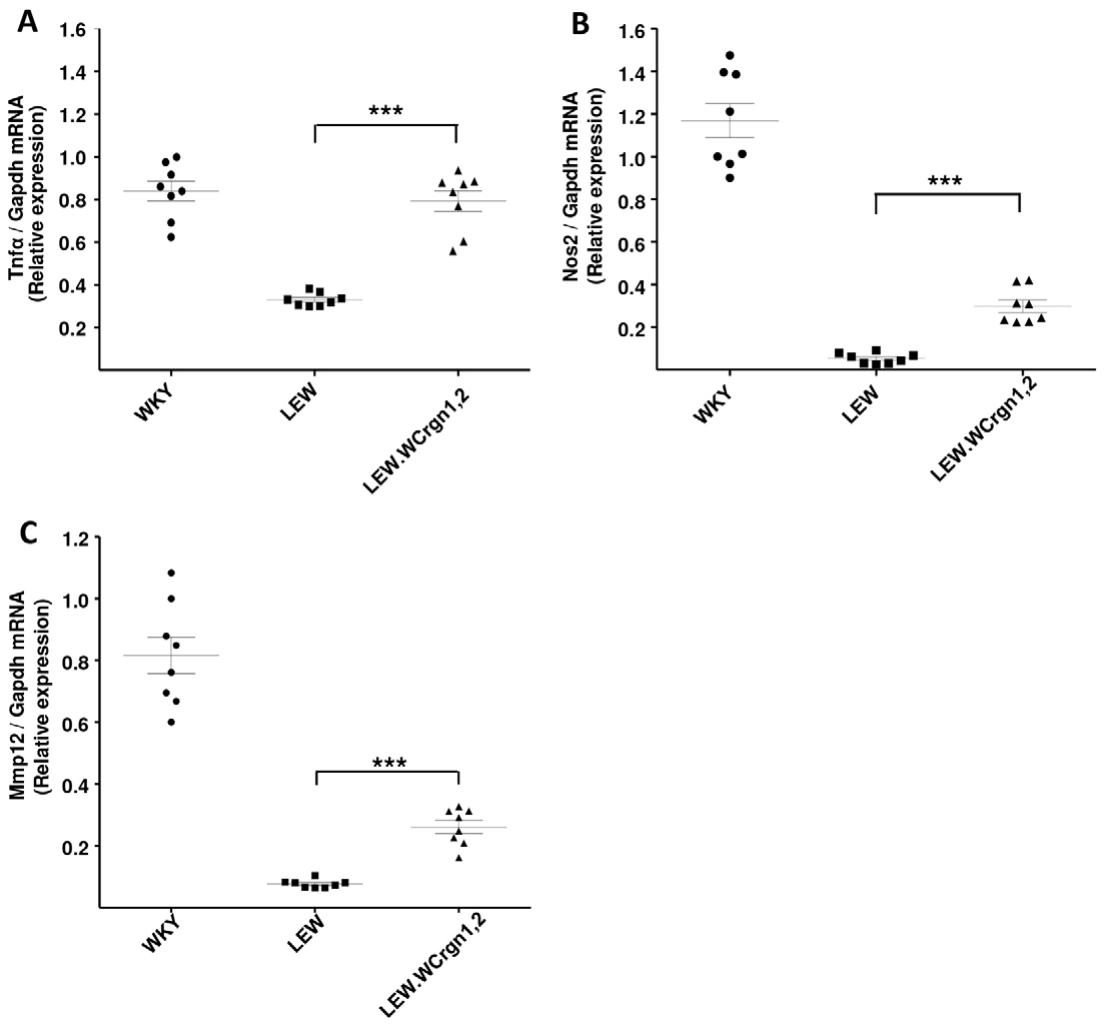
***Tnfα*,*Nos2* and *Mmp12* expression from nephritic glomeruli.** (A) Pro-inflammatory cytokine Tumour necrosis factor-α; (B) nitric oxide synthase-2; and (C) matrix metalloproteinase-12 expression measured in nephritic glomeruli 10 days after induction of NTN. Analyses carried out by qRT-PCR. ***P<0.001 between LEW.W*Crgn1,2* and LEW rats using one-way ANOVA with Newman-Keuls comparison test. *n*=4 in all strains.

The key source of the proinflammatory mediators tumour necrosis factor-α (*Tnfα*) and nitric oxide synthase 2 (*Nos2*; formerly known as inducible Nos or *iNos*) in the glomeruli is through the infiltration of macrophages (Cook et al., 1994; Tipping et al., 1991). At 10 days after the induction of NTN, nephritic glomeruli were extracted and the expression levels of glomerular *Tnfα* and *Nos2*, analysed by quantitative real-time PCR (qRT-PCR), were used to assess the extent of glomerular macrophage activation. Introgression of *Crgn1* and *Crgn2* from the CRGN-susceptible WKY into the genetic background of the CRGN-resistant LEW significantly increased glomerular *Tnfα* and *Nos2* levels compared with the parental LEW strain (Fig. 2A,B). In addition, we assessed glomerular expression of matrix metalloproteinase-12 (*Mmp12*), a protease associated with the degradation of GBM, as a readout of NTN-mediated glomerular inflammation and showed that *Mmp12* was significantly overexpressed in LEW.W*Crgn1,2* rat nephritic glomeruli when compared with parental LEW rats (Fig. 2C).

### LEW.W*Crgn1,2* rats show resistance to EAG

**Fig. 3.**
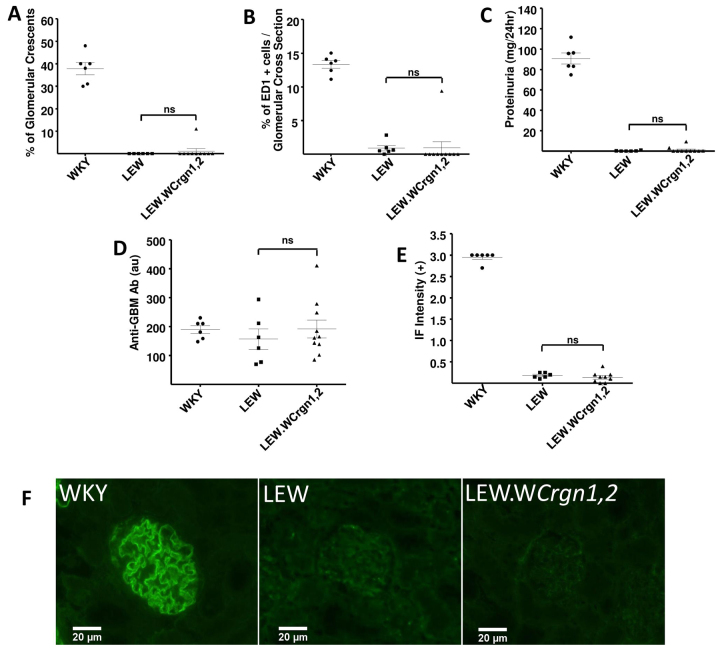
**EAG phenotypes and autoantibody responses in parental (WKY and LEW) and double congenic (LEW.W*Crgn1,2*) rat strains at day 28.** (A) Percentage of glomeruli with crescents. (B) Macrophage infiltration quantified by the percentage of ED1+ cells per glomerular cross-section. (C) Measurement of proteinuria. (D) Serum circulating anti-GBM antibody levels, showing similar serological autoimmunity in all three strains. (E) Quantification of deposited anti-GBM antibody (graded 0 to 3+ intensity) in each rat strain assessed by direct immunofluorescence (IF) for anti-rat immunoglobulins. (A–E) Statistically significant differences in mean values between the LEW.W*Crgn1,2* strain and LEW strain compared using a one-way ANOVA with Newman-Keuls comparison test. ns: non-significant. WKY and LEW rats, *n*=6; LEW.W*Crgn1,2* rats, *n*=10. (F) Representative direct immunofluorescence images showing pattern and intensity of anti-GBM antibody deposition in glomeruli in each rat strain.

EAG was induced by immunisation with recombinant rat α3(IV)NC1. Disease in this model has a more gradual onset relative to NTN, reflecting the time required for adaptive immune responses to be initiated; as a result, animals were observed for 28 days following disease induction. As previously reported (Reynolds et al., 2003), the WKY rat strain was susceptible to the induction of EAG, and all animals showed marked crescent formation, glomerular macrophage infiltration and proteinuria by day 28. The LEW strain was entirely resistant to induction of EAG, with no animals demonstrating features of disease. The LEW.W*Crgn1,2* bicongenic rat strain was also resistant, showing no significant differences from the parental LEW strain in all three phenotypic disease parameters (Fig. 3A–C). All three strains had similar levels of circulating anti-GBM antibodies (Fig. 3D), although significant levels of deposited anti-GBM antibodies, as demonstrated by direct immunofluorescence, were found in the WKY strain alone (Fig. 3E,F).

### Measurement of Fc-receptor-dependent and -independent macrophage activation in the LEW.W*Crgn1,2* strain

**Fig. 4.**
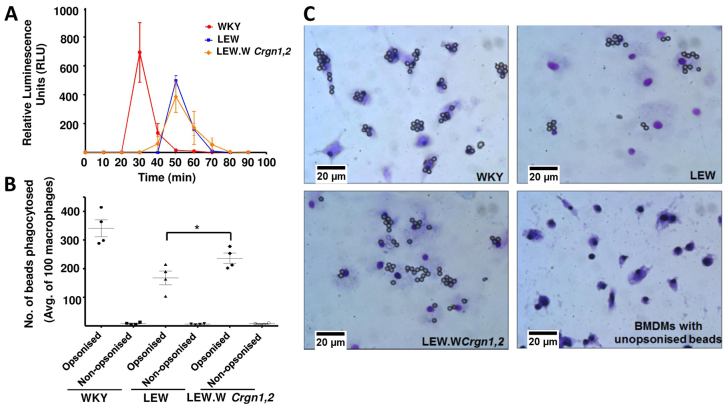
**Macrophage activation assays in BMDMs in parental (WKY and LEW) and double congenic (LEW.W*Crgn1,2*) rat strains.** (A) Production of superoxide assessed by chemiluminescence over 90 minutes, following addition of PMA (1 μM) (*n*=3 in all strains). (B,C) Fc-receptor-mediated phagocytic activity of BMDMs assessed by incubating WKY, LEW and LEW.W*Crgn1,2* BMDMs with beads opsonised with rabbit anti-BSA IgG, or unopsonised for 30 minutes. Cells were then fixed and beads in 100 BMDMs were counted (*n*=4 in all strains). *P<0.05 between LEW.W*Crgn1,2* and LEW rats using one-way ANOVA with Newman-Keuls comparison test.

To examine the combined effect of these loci on the functional phenotype of BMDMs, we carried out activation assays that could reflect the activity of BMDMs *in vivo*. Superoxide anion is a reactive oxygen species produced by phagocytes such as macrophages in response to appropriate stimuli. Levels of superoxide produced by BMDMs from WKY, LEW and LEW.W*Crgn1,2* rats were detected by chemiluminescence following stimulation with phorbol 12-myristate 13-acetate (PMA) (Fig. 4A). The kinetics of superoxide production were significantly different between the WKY versus the LEW and LEW.W*Crgn1,2* strains, because WKY BMDMs produced superoxide at an earlier time-point after stimulation compared with the LEW and LEW.W*Crgn1,2* BMDMs (Fig. 4A). There was, however, no significant difference in superoxide production between the bicongenic LEW.W*Crgn1,2* and parental LEW BMDMs. Macrophage activation was also analysed using Fc-mediated phagocytosis (Fig. 4B,C). Notably, there was a significant difference in phagocytic activity between the LEW and LEW.W*Crgn1,2* strains. These data indicate that introgressing both WKY *Crgn1* and *Crgn2* into the genetic background of the LEW strain had an effect on Fc-mediated phagocytosis but not on PMA-mediated superoxide production.

### Expression of *Nos2*, *Arg1*, *Il10*, *Lilrb3l* and *Nov* in BMDMs

**Fig. 5.**
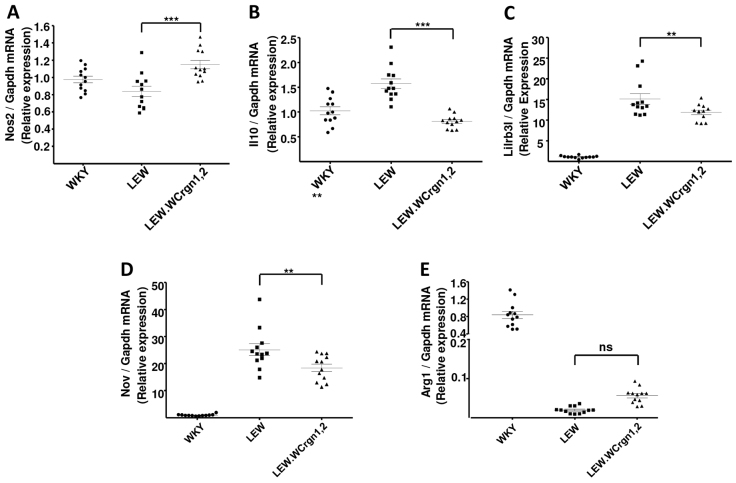
**Gene expression profiles in BMDMs in parental (WKY and LEW) and double congenic (LEW.W*Crgn1,2*) rat strains.** (A) Pro-inflammatory cytokine *Nos2*; (B) anti-inflammatory cytokine *Il10*; (C) *Lilrb3l*; (D) *Nov* and (E) *Arg1* expression in LPS-stimulated BMDMs (except *Nov*, for which BMDMs in basal state). Expression analyses were carried out by qRT-PCR. ***P<0.001; **P<0.01; *P* was non-significant (ns) between LEW.W*Crgn1,2* and LEW rats using one-way ANOVA with Newman-Keuls comparison test. *n*=4 biological rats per strain with three technical replicates per rat. BMDMs were untreated (basal) or stimulated with 100 ng LPS for 4 hours.

Macrophages have been broadly classified according to their activation phenotypes as M1 or M2 macrophages. M1 macrophages are capable of secreting proinflammatory mediators such as cytokines and are strongly microbicidal, whereas M2 macrophages secrete anti-inflammatory cytokines, promoting wound-healing, immune regulation and resolution of inflammation (Mosser and Edwards, 2008). We have previously determined the macrophage transcriptome of NTN-susceptible WKY and NTN-resistant LEW rats by identifying ∼700 differentially expressed transcripts between the BMDMs of the two strains (Maratou et al., 2011). To examine the effect of LEW *Crgn1* and *Crgn2* on the macrophage transcriptome, we assessed the expression of M1 and M2 markers (*Nos2* and *Il10*, respectively), as well as the most robustly differentially expressed transcripts (*Arg1*, *Lilrb3l* and *Nov*) between WKY and LEW BMDMs. Analysis of expression levels was carried out by qRT-PCR of mRNA from BMDMs either stimulated with lipopolysaccharide (LPS, 100 ng, 4 hours) (*Nos2*, *Il10*, *Arg1*, *Lilrb3l*) or in the basal state (*Nov*). There was significantly enhanced expression of the proinflammatory cytokine *Nos2* in LEW.W*Crgn1,2* BMDMs compared with the CRGN-resistant LEW parental strain (Fig. 5A). The presence of *Crgn1* and *Crgn2* in LEW.W*Crgn1,2* rats significantly lowered the expression of the anti-inflammatory cytokine *Il10* to levels similar to that in the CRGN-susceptible WKY strain (Fig. 5B). Introgression of *Crgn1* and *Crgn2* also caused a reduction in the levels of *Lilrb3l* and *Nov* (Fig. 5C,D), and conversely showed an increased trend in levels of *Arg1* in BMDMs from LEW.W*Crgn1,2* rats compared with expression levels from parental LEW BMDMs in the same condition (Fig. 5E).

## DISCUSSION

GN is a leading cause of human kidney failure, but the underlying pathogenesis is incompletely understood. The WKY model has contributed substantially to understanding the molecular basis of CRGN, with identification of *Fcgr3* and *Jund* as susceptibility genes (Aitman et al., 2006; Behmoaras et al., 2008). We report here the generation and phenotypic characterisation of a new rat model of CRGN, on the normally resistant LEW genetic background. We replaced the LEW alleles for *Fcgr3* and *Jund* with the corresponding WKY alleles, creating a new bicongenic strain, LEW.W*Crgn1,2*, and found that this strain is susceptible to NTN, including the development of glomerular crescents, but is resistant to the related CRGN phenotype EAG. Characterisation of cytokine profiles and functional testing of the LEW.W*Crgn1,2* glomeruli and macrophages provide preliminary insights into the genes and pathways leading to development of CRGN in this new model.

Our earlier research focussed on the genetic dissection of CRGN in segregating populations derived from the CRGN-susceptible and CRGN-resistant rat strains, WKY and LEW, respectively. The two CRGN susceptibility loci, *Crgn1* and *Crgn2*, identified by genome-wide linkage analysis and functional studies, showed the deletion of *Fcgr3-rs* to be the cause of macrophage overactivity at *Crgn1* (Aitman et al., 2006), and, subsequently, the marked upregulation of *Jund* at *Crgn2* was associated with increased macrophage activation (Behmoaras et al., 2008; Hull et al., 2013).

In addition, we have described the effects of NTN on a double congenic rat strain for chromosomes 13 and 16 (WKY.L*Crgn1,2*) generated by introgressing LEW *Crgn1* and *Crgn2* into the genetic background of the WKY strain. The LEW *Crgn1* and *Crgn2* loci conferred marked additive protective effects on NTN phenotypes in WKY.L*Crgn1,2* rats, as seen by a significant reduction in glomerular crescent formation, macrophage infiltration and proteinuria compared with parental WKY rats. The WKY.L*Crgn1,2* strain also showed reduced levels of glomerular cytokines, protease production and *Nos2*, as well as reduced macrophage activation (Behmoaras et al., 2010). Although this study by Behmoaras et al. allowed the extent of the roles of LEW *Crgn1* and *Crgn2* in CRGN susceptibility to be examined, the effects of WKY *Crgn1* and *Crgn2* on a LEW genetic background, in NTN or EAG studies, were sofar unknown.

In this study, we report a new bicongenic rat strain, LEW.W*Crgn1,2*, in which the CRGN-susceptible WKY loci (*Crgn1* and *Crgn2*) have been introgressed into a CRGN-resistant LEW genetic background. We investigated susceptibility of this new LEW.W*Crgn1,2* strain to two distinct models of macrophage-dependent CRGN (NTN – requiring passive transfer of heterologous nephrotoxic antibodies raised in another species – and EAG – requiring induction of autoimmunity to the GBM) compared with the LEW strain. We found that the presence of these WKY CRGN loci in the LEW.*WCrgn1,2* strain led, for the first time, to significant crescent formation, infiltration of macrophages and proteinuria, after a single dose of NTS, in a rat strain with a LEW genetic background. It is important to highlight that the LEW rat strain has been used consistently in NTN studies as a robustly reproducible negative control (Aitman et al., 2006; Behmoaras et al., 2008; Behmoaras et al., 2010; Hull et al., 2013; Maratou et al., 2011; Page et al., 2012; Smith et al., 2007). Our findings demonstrate that, when the two CRGN susceptibility loci (*Crgn1* and *Crgn2*) from the WKY strain are introgressed into the LEW strain, this CRGN-resistant strain develops glomerular crescents, a hallmark of CRGN, and that the presence of both *Crgn1* and *Crgn2* are essential for the development of disease.

Macrophages are well recognised mediators of glomerular injury in CRGN (Cattell, 1994; Cook et al., 1999; Duffield, 2010; Isome et al., 2004; Williams et al., 2010), and the importance of macrophage number and activity within the glomerulus for crescent formation and disease progression has been previously established in rat models of CRGN (Behmoaras et al., 2010; Isome et al., 2004; Munger et al., 1999). The presence of the proinflammatory cytokines *Tnfα* and *Nos2* in the glomerulus comes largely from infiltrating macrophages (Cook et al., 1994; Tipping et al., 1991). Furthermore, *Mmp12*, which is secreted by macrophages, enables degradation of extracellular matrix components, such as those of the GBM (Kaneko et al., 2003). We found that the expression of *Tnfα*, *Nos2* and *Mmp12* was significantly increased in the nephritic glomeruli of LEW.W*Crgn1,2* rats compared with parental LEW rats. Interestingly, levels of nephritic glomerular *Tnfα* in the LEW.W*Crgn1,2* rats were comparable with those in WKY rats, despite a significant difference in infiltrating macrophage numbers into the glomeruli between the two strains, suggesting that glomerular *Tnfα* levels might indicate macrophage activation rather than infiltration.

In order to assess and compare the functional phenotype of macrophages from the WKY, LEW and bicongenic LEW.W*Crgn1,2* strains, we carried out two tests, namely, a superoxide anion detection assay and an Fc-mediated bead phagocytosis assay. Superoxide, a reactive oxygen species, is produced by activated phagocytes, such as macrophages, as part of their biological defence mechanism (Babior et al., 1973; Robinson, 2009). *In vitro*, macrophages can be stimulated to generate superoxide by PMA. PMA, a phorbol ester, is a potent inflammatory and tumour-producing compound, capable of mimicking diacylglycerol, thereby activating the protein kinase C signalling pathway (Castagna et al., 1982; Driedger and Blumberg, 1980; White et al., 1984). The overproduction of superoxide often leads to tissue injury at sites of inflammation (Halliwell and Gutteridge, 2006). Furthermore, superoxide produced by macrophages has been reported to be a prominent contributor to glomerular injury in glomerulonephritis (Cook et al., 1989). Here, we measured superoxide produced by BMDMs from WKY, LEW and LEW.*WCrgn1,2* rats by chemiluminescence. Although the amount and the kinetics of superoxide production differed significantly between the WKY and LEW BMDMs, there was no difference between the LEW and the LEW.W*Crgn1,2* BMDMs, indicating that WKY *Crgn1* and *Crgn2* had no influence on the protein kinase C signalling pathway, when introgressed into a LEW genetic background.

Fc receptors for IgG (Fcgr) have been shown to facilitate phagocytosis and antibody-dependent cellular cytotoxicity in rodent macrophages (Aitman et al., 2006; Behmoaras et al., 2008; Behmoaras et al., 2010; Mosser and Zhang, 2008). We have previously shown that the absence of an Fc-related sequence (*Fcgr3-rs*) causes macrophage overactivity and NTN susceptibility in the WKY strain, whereas, conversely, the LEW strain, which has *Fcgr3-rs*, is resistant to NTN and shows no macrophage overactivity (Aitman et al., 2006; Page et al., 2012). Consistent with this, Fc-mediated bead phagocytosis levels were higher in WKY BMDMs than in LEW BMDMs. Notably, by introgressing the WKY *Fcgr3* gene in the *Crgn1* locus, along with *Crgn2*, into the genetic background of the LEW strain, we observed an increase in phagocytosis in LEW.W*Crgn1,2* BMDMs, reflecting macrophage activation in this strain brought about by the synergistic influence of *Crgn1* and *Crgn2.*

In the NTN model, *Crgn1* and *Crgn2* exert their influence chiefly on bone-marrow cells, specifically BMDMs, rather than on intrinsic renal cells (Behmoaras et al., 2010). Because of this, we analysed the effects of WKY *Crgn1* and *Crgn2* in LEW.W.*Crgn1,2* rats on BMDM gene expression and found that these two CRGN susceptibility loci caused a significant increase in expression of the proinflammatory cytokine *Nos2*, and conversely a reduction in expression of the anti-inflammatory cytokine *Il10*, when compared with parental LEW BMDMs. Our previous work characterised macrophage transcriptomes of the WKY and LEW strains, which revealed highly significant differentially expressed genes between WKY and LEW BMDMs (in basal or LPS-stimulated states) that are in effector pathways for macrophage-mediated damage in CRGN (Maratou et al., 2011). We found that, in LEW.W*Crgn1,2* BMDMs, there was a significant decrease in expression levels of genes that are normally overexpressed in LEW BMDMs (*Lilrb3l*, *Nov*) and conversely an increasing trend of *Arg1* expression (normally overexpressed in WKY BMDMs) in LEW.W*Crgn1,2* BMDMs compared with LEW BMDMs. This correlates with the important effects exerted by WKY *Crgn1* and *Crgn2* on the LEW genome, whereby the introgression of these CRGN susceptibility QTLs causes the LEW BMDMs to have a similar phenotype to that of the WKY strain.

In contrast to NTN, the bicongenic LEW.W*Crgn1,2* strain was resistant to EAG. Interestingly, previous work by our group has shown that WKY.L*Crgn1* single congenic animals show significant protection from disease in EAG (Reynolds et al., 2012). The failure to confer susceptibility to EAG in the bicongenic LEW.W*Crgn1,2* strain, however, confirms our previous observation that susceptibility to EAG has a different mode of inheritance to NTN (Reynolds et al., 2002), with likely roles for additional susceptibility loci. It is intriguing that both LEW.W*Crgn1,2* and parental LEW rats make similar levels of circulating anti-GBM antibodies to WKY rats, but do not show significant deposition on the GBM. We can hypothesise a number of possible reasons. Firstly, it might be that the antibodies differ between strains in terms of epitope specificity, affinity or IgG subclass. Secondly, there might be differences in accessibility of the epitopes in the GBM between strains, perhaps related to concomitant T-cell-mediated injury in the WKY strain; however, anti-GBM antibodies eluted from the kidneys of WKY rats can bind to the GBM of LEW rats (Reynolds et al., 2012). The failure of the bicongenic LEW.W*Crgn1,2* strain, like LEW, to develop deposited autoantibody in the glomerulus suggests that EAG susceptibility is most likely under the influence of additional WKY loci that control glomerular autoantibody deposition. These loci must reside outside the rat MHC, because WKY and LEW strains share a common RT-1 haplotype (RT1^l^). The identification of these additional loci that confer protection in LEW and LEW-related strains, yet susceptibility in the WKY strain, is the subject of our ongoing work.

Our future work aims to investigate the effect of WKY *Crgn1* and *Crgn2* in the bicongenic strain across the entire transcriptome. The LEW.W*Crgn1,2* strain will also provide the basis for future work to define the mechanisms underlying the progression from serological autoimmunity (which is shown in the LEW.W*Crgn1,2* strain), through glomerular autoantibody deposition (absent in the LEW.W*Crgn1,2* strain), to glomerular inflammation (absent in the LEW.W*Crgn1,2* strain, but to which it is critically susceptible, unlike the parental LEW strain).

In conclusion, our study has shown, for the first time, the formation of glomerular crescents in NTN in a rat strain with a LEW genetic background, which is normally resistant to NTN. Despite showing susceptibility to NTN, the new bicongenic LEW.W*Crgn1,2* strain is resistant to glomerular autoantibody deposition and to EAG. These data emphasise the importance of *Crgn1* and *Crgn2* on NTN susceptibility and show the potential value of the LEW.W*Crgn1,2* strain for future studies of macrophage-dependant glomerulonephritis.

## MATERIALS AND METHODS

### Generation of congenic rat strains

Parental WKY (WKY/NCrl) and LEW (LEW/Crl) rats were purchased from Charles River. Generation of the bicongenic rat strain, LEW.W*Crgn1,2*, was achieved as follows: single congenic strains LEW.W*Crgn1* and LEW.W*Crgn2* (Behmoaras et al., 2008) were crossed to produce an F_1_ population that was then backcrossed to LEW.W*Crgn1*. Backcross animals that were heterozygous for *Crgn2* and homozygous for *Crgn1* were selected and crossed by brother-sister mating to obtain animals that were bicongenic for WKY *Crgn1* and *Crgn2* on a LEW genetic background. The congenic intervals and microsatellite markers used for genotyping were as described previously (Behmoaras et al. 2010), with the difference being the reversal of donor and recipient strains used in the present study. All procedures were performed in accordance with the United Kingdom Animals (Scientific Procedures) Act, 1986.

### NTN and nephritic glomeruli isolation

NTS was prepared as previously described (Bhan et al., 1978). NTN was induced in 8-week-old male WKY, LEW, LEW.W*Crgn1*, LEW.W*Crgn2* and LEW.W*Crgn1,2* rats by intravenous injection of 0.1 ml of NTS. Nine days later, urine was collected by placing rats in metabolic cages for 24 hours with free access to food and water. Proteinuria was determined by the sulphosalicylic acid test (Baker et al., 1998). Ten days after NTN induction, rats were culled by asphyxiation with CO_2_ and cervical dislocation, glomeruli were isolated from one kidney each from all strains (four rats per strain) while the other kidney from all strains was formalin-fixed and paraffin embedded. Glomerular isolation was carried out as described (Deplano et al., 2013). Glomerular pellets were then resuspended in TRIzol^®^ (Invitrogen).

### Induction and assessment of EAG

Six-week-old female WKY, LEW and LEW.W*Crgn1,2* rats were immunized with 0.5 mg/kg recombinant rat α3(IV)NC1 in complete Freund’s adjuvant by intramuscular injection. After 28 days, urine, serum and tissues were collected and proteinuria determined as described for NTN animals. Serum anti-GBM antibody concentrations were assayed as described previously (Ryan et al., 2001). Glomerular deposition of anti-GBM antibodies was detected by direct immunofluorescence (Reynolds et al., 2012).

### Histology and immunohistochemistry

To quantify the degree of histological injury in both the NTN and EAG models, 4 μm formalin-fixed paraffin-embedded kidney sections were stained with H&E and periodic acid-Schiff (PAS). 100 consecutive glomeruli were assessed in a blinded manner, and the number of severely crescentic glomeruli expressed as a percentage of total glomeruli examined. To quantify the number of macrophages that infiltrated into glomeruli, formalin-fixed paraffin-embedded kidney sections were stained with mouse monoclonal antibody to ED-1 (Serotec, Oxford, UK), followed by an HRP-labelled anti-mouse polymer development system (Dako Ltd, UK). The cellular infiltrate in 20 consecutive glomeruli was quantified using automated image analysis software (ImagePro Plus, Media Cybernetics, Bethesda, MD) and expressed as a percentage of total glomerular cross-sectional area.

### BMDM culture, bead phagocytosis and superoxide anion detection

BMDMs were obtained and characterised as described previously (Behmoaras et al., 2010). Bone-marrow cells were allowed to differentiate in Dulbecco’s modified Eagle’s medium (DMEM; Gibco) containing 25 mM HEPES buffer (Sigma), 25% L929-conditioned medium, 25% fetal bovine serum (Biosera), penicillin (100 U/ml; Gibco) and streptomycin (100 μg/ml; Gibco), and cultured for 5 days on Petri dishes (Nunc).

Macrophage phagocytosis was assessed as described (Behmoaras et al., 2008; May et al., 2000). Dissociated day-5 BMDMs were allowed to adhere overnight to eight-well chamber slides (Nunc) at a cell density of 10^5^ cells per chamber. After addition of 6-μm polystyrene beads (Polysciences), unopsonised or opsonised with rabbit anti-BSA IgG (Sigma) (Behmoaras et al., 2008), the chamber slides were incubated for 30 minutes at 37°C, 5% CO_2_, followed by a wash with 1× PBS and fixed with Diffquick (Medion Diagnostics).

The reactive oxygen species, superoxide anion, was assessed by a chemiluminescence assay (LumiMax Superoxide Anion Detection Kit, Stratagene), wherein relative luminescence units (RLU) corresponded to superoxide levels produced by BMDMs. Briefly, day-5 BMDMs were dissociated using cell dissociation solution (Sigma) and allowed to adhere overnight to a 96-well optical bottom plate (Nunc) at a cell density of 2.5×10^5^ cells per well with BMDMs plated from four rats/strain in triplicate. Prior to the assay, cells were washed and PMA (1 μM; Sigma) was used to generate superoxide production by BMDMs. A time-dependent increase in chemiluminescence was detected using the Fluostar Galaxy plate reader (BMG Labtech) and the RLU values were detected for a total period of 100 minutes.

### RNA extraction and real-time quantitative RT-PCR

Total RNA was extracted from nephritic glomeruli and BMDMs using the TRIzol^®^ method, which was then quantified using the NanoDrop^®^ND-1000 Spectrophotometer (Thermo Scientific). Real-time quantitative RT-PCR was carried out using the 7500 Fast Real Time PCR System (Applied Biosystems) and the Brilliant^®^ SYBR^®^ Green QRT-PCR kit (Agilent Technologies). 100 ng of total RNA was utilised for qRT-PCR, with each sample amplified in duplicate. Samples were first subjected to reverse transcription (30 minutes at 50°C and 10 minutes at 95°C) followed by cycling 40 times at 95°C for 15 seconds and 60°C for 1 minute. Primer sequences are available upon request. Results obtained were exported to the 7500 Fast System SDS software (Applied Biosystems), where C_t_ values were determined and normalised to glyceraldehyde-3-phosphate dehydrogenase gene expression. Relative expression levels were then determined using the 2^−ΔΔCt^ method.

### Statistical analysis

Results are expressed as mean ± s.e.m. Statistical differences in mean values between the bicongenic LEW.W*Crgn1,2* strain and parental LEW rats were compared using a one-way ANOVA with Newman-Keuls comparison test.

### Deposition of resource in a repository

The new bicongenic LEW.W*Crgn1,2* rat strain will be available as a resource from the National BioResource Project (NBRP) – Rat, Kyoto University, Kyoto Japan.
